# Developing cancer therapies – think global

**DOI:** 10.18632/aging.101129

**Published:** 2016-11-16

**Authors:** Andrew D. Hollenbach

**Affiliations:** Department of Genetics, Louisiana State University Health Sciences Center, New Orleans, LA 70112, USA

**Keywords:** Alveolar Rhabdomyosarcoma, pathway analysis, multi-faceted treatment regimen, cancer therapies

Cancer is a disease that afflicts individuals regardless of age. It may occur in children, adolescents, or young adults who are born with genetic mutations that predispose them to the disease. It also occurs in the elderly where a lifetime of exposure to environmental or age-related stressors facilitates the acquisition of oncogenic mutations. Regardless of the age of onset, cancer is a multifaceted and deeply complex disease that results from the accumulation of multiple genetic alterations that allow cells to evade normal regulatory safeguards to develop into a metastatic tumor.

Many times the cascade of genetic alterations leading to the metastatic state is initiated by a single driver mutation, a single genetic event that facilitates or promotes the generation of subsequent mutations culminating in the oncogenic state. In a recent publication we identified a known oncogenic protein to be one such driving mutation [1]. Alveolar Rhabdomyosarcoma (ARMS), an aggressive pediatric muscle tumor, is primarily characterized by the somatically acquired t(2;13)(q35;q14) balanced chromosomal translocation, which generates the oncogenic fusion protein PAX3-FOXO1. We determin-ed that the sole expression of PAX3-FOXO1 is sufficient to promote aneuploidy and overcome aneuploidy-dependent cell cycle arrest in the progression to the oncogenic state, thereby serving as a driver mutation in ARMS. More importantly, we showed that PAX3-FOXO1 achieves this by globally altering mRNA and miRNA expression levels to negatively affect all aspects of chromosomal segrega-tion and stability (leading to aneuploidy) while positively affecting at least five different growth factor related signaling pathways (leading to overcoming proliferative arrest).

Given the global nature of PAX3-FOXO1 induced expression changes (in which over 100 genes are directly or indirectly affected to promote aneuploidy and proliferative effects), it is not surprising that present experimental therapeutic strategies are proving ineffective in clinical trials. Many of these therapies focus on inhibiting a single gene or pathway located genetically downstream of the oncogenic fusion protein. However, our results highlight the fact that ARMS tumor cells have altered the expression of multiple genes involved in redundant biological processes (e.g., growth factor related proliferation). As such ARMS tumor cells can easily compensate for the loss or inhibition of a single gene or pathway under present experimental therapeutic approaches. Based on this information we proposed the development of a multifaceted treatment regimen in which we suggest using small molecules to inhibit three essential biological pathways important for the development of ARMS: 1) prevent phosphorylation of PAX3-FOXO1 at the key regulatory site; 2) kill aneuploid cells; and 3) inhibit at least one of the affected growth factor related pathways.

How then do these results, results that allow us to describe potential treatment therapies for a pediatric tumor with a known driver genetic mutation, have implications for an ageing patient diagnosed with a tumor with no explicitly known driving genetic mutation? It is true that many individual mutations are known to play important roles in the development of cancer in the ageing population (e.g., KRAS or p53). However, it is also known that a complex series of mutations that affect global gene and miRNA expression contribute to the development and progression of a normal cell to the metastatic state. Although this complexity results from a large heterogeneity in the nature of the individual mutations and gene expression changes, these individual changes usually converge to affect targetable biological pathways, which in the case of ARMS is aneuploidy, phosphorylation, and growth factor related pathways.

Therefore, in my opinion we as researchers should “think globally” and attempt to identify how the complexity of mutations and expression changes work together to produce a biological “signature” of pathway alterations in individual tumor types. Present day technology allows the relatively rapid sequencing of genomes, transcriptomes, microRNAomes, and determination of methylation patters, which can easily identify the global nature of mutations and expression changes in tumors relative to normal tissue in individual patients. Subsequent bioinformatics analyses can then identify biological pathways and processes affected by these changes, which when compared across multiple individual patient samples within a tumor type is likely to produce a biological “signature” for that particular cancer. These signatures could ultimately elucidate key biological targets for use in developing multi-faceted regimens, as we described for ARMS.
